# Long non-coding RNA LINC00680 functions as a ceRNA to promote esophageal squamous cell carcinoma progression through the miR-423-5p/PAK6 axis

**DOI:** 10.1186/s12943-022-01539-3

**Published:** 2022-03-07

**Authors:** Song-tao Xue, Bin Zheng, Shi-qiang Cao, Jian-cheng Ding, Guo-sheng Hu, Wen Liu, Chun Chen

**Affiliations:** 1 Department of Thoracic Surgery, Fujian Medical University Union Hospital, No. 29 Xinquan Road, Fuzhou, 350001 Fujian China; 2 Fujian Key Laboratory of Cardio-Thoracic Surgery, Fujian Medical University, No. 29 Xinquan Road, Fuzhou, 350001 Fujian China; 3 State Key Laboratory of Cellular Stress Biology, School of Pharmaceutical Sciences, Xiamen University, Xiang’an South Road, Xiamen, 361102 Fujian China; 4 Fujian Provincial Key Laboratory of Innovative Drug Target Research, School of Pharmaceutical Sciences, Xiamen University, Xiang’an South Road, Xiamen, 361102 Fujian China

**Keywords:** ESCC, LncRNA, CeRNA, LINC00680, miR-423-5p, PAK6, ASO

## Abstract

**Background:**

Esophageal squamous cell carcinoma (ESCC) is a common invasive malignancy worldwide with poor clinical outcomes. Increasing amount of long non-coding RNAs (lncRNAs) have been reported to be involved in cancer development. However, lncRNAs that are functional in ESCC and the underlying molecular mechanisms remain largely unknown.

**Methods:**

Transcriptomic analysis was performed to identify dysregulated lncRNAs in ESCC tissue samples. The high expression of LINC00680 in ESCC was validated by RT-qPCR, and the oncogenic functions of LINC00680 was investigated by cell proliferation, colony formation, migration and invasion assays in ESCC cells in vitro and xenografts derived from ESCC cells in mice. RNA-seq, competitive endogenous RNA (ceRNA) network analysis, and luciferase reporter assays were carried out to identify LINC00680 target genes and the microRNAs (miRNAs) bound to LINC00680. Antisense oligonucleotides (ASOs) were used for in vivo treatment.

**Results:**

Transcriptome profiling revealed that a large number of lncRNAs was dysregulated in ESCC tissues. Notably, LINC00680 was highly expressed, and upregulation of LINC00680 was associated with large tumor size, advanced tumor stage, and poor prognosis. Functionally, knockdown of LINC00680 restrained ESCC cell proliferation, colony formation, migration, and invasion in vitro and inhibited tumor growth in vivo. Mechanistically, LINC00680 was found to act as a ceRNA by sponging miR-423-5p to regulate PAK6 (p21-activated kinase 6) expression in ESCC cells. The cell viability and motility inhibition induced by LINC00680 knockdown was significantly reversed upon PAK6 restoration and miR-423-5p inhibition. Furthermore, ASO targeting LINC00680 substantially suppressed ESCC both in vitro and in vivo.

**Conclusions:**

An oncogenic lncRNA, LINC00680, was identified in ESCC, which functions as a ceRNA by sponging miR-423-5p to promote PAK6 expression and ESCC. LINC00680/miR-423-5p/PAK6 axis may serve as promising diagnostic and prognostic biomarkers and therapeutic targets for ESCC.

**Supplementary Information:**

The online version contains supplementary material available at 10.1186/s12943-022-01539-3.

## Introduction

Esophageal squamous cell carcinoma (ESCC) is one of the most commonly diagnosed malignancies, and it is the sixth leading cause of cancer-related death worldwide, depriving more than 400,000 patients of their lives each year [1, 2]. Despite remarkable improvements in diagnosis and treatment, the five-year survival rate of ESCC patients remains below 20% [3, 4]. A more thorough understanding of the molecular mechanisms, and developing effective diagnostic and prognostic methods is urgently needed for ESCC.

In human genome, there is only up to 2% of protein-coding genes accounts for stable transcription, whereas the vast majority are non-coding RNAs (ncRNAs) [5, 6]. Growing reports have shown that ncRNAs, especially long non-coding RNAs (lncRNAs), play vital roles in various types of cancers via mediating coding gene expression at transcriptional, posttranscriptional, and/or translational levels. LncRNAs, by definition, are a form of ncRNAs greater than 200 nt in length, which have drawn increasing attentions for their participation in the development of cancers [7–11]. The aberrant expression of lncRNAs has been frequently observed in various types of cancers including ESCC, and act as tumor suppressors or drivers via influencing diverse cellular malignant processes, such as cell proliferation, apoptosis, cell cycle, migration, invasion, and drug resistance [12–16]. For instance, lncRNA ATB is found to be dysregulated in ESCC and competitively bind to the miR-200 family to promote ESCC cell proliferation, migration, invasion, and EMT progression through upregulating Kindlin-2 expression [17]. LncRNA ESCCAL-1 exerted an oncogenic function in ESCC by positively regulating malignant behaviors of cancer cells during ESCC development via sponging miR-590-3p to modulate the expression of APOBEC3G [18]. THAP9-AS1 facilitated ESCC progression through a positive feedback loop constituting of THAP9-AS1/miR-133b/SOX4 axis [19]. LncRNA KLF3-AS1 expression was suppressed in ESCC and contributed to the enhancement of the invasive capacity of ESCC cells by impairing miR-185-5p-mediated inhibition of KLF3 [20]. However, regulatory mechanisms for lncRNA-dependent gene expression in ESCC deserve in-depth exploration in order to develop promising therapeutic methods.

LINC00680 is annotated as a lncRNA located on 6p11.2 and has been reported to play a crucial role in human cancers including sarcoma, hepatocellular carcinoma, glioblastoma, lung adenocarcinoma, and non-small cell lung cancer [21–24]. Nevertheless, its functions and pathological mechanisms in ESCC progression remain uncharacterized. MicroRNA (miRNA) is a class of shorter non-coding RNAs that regulating gene expression largely by binding to the 3ʹ-untranslated region (3ʹUTR) of target protein-coding genes. A model of lncRNA involvement in gene regulation, called competing endogenous RNA (ceRNA), has been proposed that lncRNAs affect mRNA expression by competitively binding with miRNAs [25–27].

Antisense oligonucleotides (ASOs) are short, single-stranded, synthetic analogues of natural nucleic acids designed to specifically bind to the complementary RNA in a sequence-specific manner in both nucleus and cytosol of cells [28–30]. Importantly, ASOs can be designed to target genes associated with diseases including cancer [31–36], which makes ASO a highly promising therapeutic strategy in clinics.

In the current study, we found that LINC00680 was significantly upregulated in ESCC and associated with poor prognosis. It was positively correlated with tumor size, depth of invasion, lymph node metastasis, and TNM stage. Silencing of LINC00680 suppressed ESCC cell proliferation, colony formation, migration, and invasion in vitro and tumor growth in mice. Mechanistically, LINC00680 functions as a molecular sponge of miR-423-5p to promote the expression of its target PAK6, modulating the progression of ESCC. Of significance, targeting LINC00680 by ASO was effective in suppressing tumor growth in mice. Overall, our findings provided a potential diagnostic biomarker and therapeutic target for ESCC patients.

## Materials and methods

### Clinical tissue specimens

Fresh tumor tissues and corresponding adjacent normal esophageal epithelial tissues were obtained from ESCC patients, who were not given any radiotherapy or chemotherapy before esophagectomy at the Department of Thoracic Surgery of Fujian Medical University Union Hospital between 2015 and 2018. After surgical resection, all specimens were placed in liquid nitrogen immediately and stored at − 80 °C until RNA extraction. All clinicopathological diagnoses were confirmed by two experienced pathologists according to the eighth edition of the American Joint Commission on Cancer (AJCC) and the Union for International Cancer Control (UICC). The ethical consent was granted by the Ethics Committee of the Fujian Medical University Union Hospital and informed consents were obtained from all patients.

### Cell culture

The human ESCC cell lines (KYSE140, KYSE150, KYSE510, EC109, and EC9706) and human normal esophageal epithelial cell line (Het-1A) were preserved by our laboratory for years and were maintained in RPMI 1640 (Biological Industries) medium with 10% fetal bovine serum (Biological Industries) and 1% penicillin/streptomycin mixture (Biological Industries) as supplements. All cell lines were cultured in a humidified incubator containing 5% CO_2_ at 37 °C.

### RNA isolation, reverse transcription, and RT-qPCR assay

The total RNA was extracted with TRIzol reagent (Takara) according to the manufacturer’s instructions. For mRNAs and lncRNAs, reverse transcription was carried out using GoScript™ Reverse Transcription Mix (Promega) with random primers. For miRNA analysis, a Bulge-Loop™ miRNA qRT-PCR primers set (one RT primer and a pair of qRT-PCR primers for each set) specific for miR-423-5p was designed and synthesized (RiboBio). RT-qPCR analysis was performed using Hieff® qPCR SYBR Green Master Mix (Yeasen) and AriaMx Real-Time PCR machine (Agilent Technologies). Actin and U6 were used as internal controls and all reactions were repeated in three independent experiments. The expression of genes measured was normalized to endogenous controls, and the relative quantification (2^−ΔΔCt^) method was used for fold-change calculation. The primers were listed in Additional file 2: Table S1.

### RNA sequencing (RNA-Seq) and computational analysis of RNA-Seq data

Ten pairs ESCC tumor and adjacent normal tissues were subjected to RNA extraction and sample preparation. Total RNA isolation was performed by using Trizol (Takara) followed by Dnase I digestion to remove residual DNA. RNA library preparation was performed by using NEBNext® Ultra™ Directional RNA Library Prep Kit for Illumina (E7420L). Paired-end sequencing was performed with Illumina NovaSeq 6000. Sequencing reads were aligned to hg38 reference genome. Cufflinks was used to quantify the expression of RefSeq annotated genes with the option -M (reads aligned to repetitive regions were masked) and -u (multiple aligned read are corrected using ‘rescue method’) [37]. Genes with FPKM (fragments per kilobase per million mapped reads) larger than or equal to 0.5 in any one of the experimental conditions were included in our analysis. FPKM of a gene was calculated as mapped reads on exons divided by exonic length and the total number of mapped reads. DESeq2 was used to determine differentially expressed genes [38]. For differentially expressed genes in ESCC clinical samples and si LINC00680-transfected ESCC cells, a cutoff of q value (for clinical samples) less than 0.05 or *P* value less than 0.001 (for si LINC00680-transfected ESCC cells) and fold change larger than 1.5 was applied. Box plot and heat map were generated by R software and significance was determined using Student’s t-test. Gene Ontology (GO) and Kyoto Encyclopedia of Genes and Genomes (KEGG) pathway analysis were performed using Metascape [39].

### Rapid amplification of cDNA ends (RACE)

The 5’ and 3’ RACE were performed using SMARTer RACE 5’/3’ Kit (Takara) according to the manufacturer’s instructions. RNA was extracted from KYSE140 cells. The gene specific primers (GSP) and nested gene specific primers (NGSP) used for 5’ and 3’ RACE were listed in Additional file 2: Table S1.

### Molecular cloning

Vector expressing wild-type LINC00680 (WT), LINC00680 with miR-423-5p binding site mutated (MT), and PAK6 were synthesized (Mailgene, China) and then cloned into the pBOBI-CS2-C-Flag or pCDH-CMV-Puro expression vector. Small hairpin RNAs (shRNA) targeting LINC00680 were synthesized (Bioray Biotechnology, China), annealed and cloned into pLKO.1 vector. shRNA targeting sequences were shown in Additional file 2: Table S1.

### Cell transfection, lentivirus packaging and infection

siRNAs, miRNA mimics, miRNA inhibitors, and ASOs as well as the matched negative controls were designed and synthesized by RiboBio (Guangzhou, China). ASO has 10 DNA nucleotides in the middle and 5 RNA nucleotides at the both ends. RNA is 2-methoxy-modified, and the thiophosphoric acid modification is between every nucleotide. ASO was also modified by cholesterol at the 5’ end when used for in vivo treatment. Lipofectamine™2000 reagent (Invitrogen, USA) was used to transfect siRNAs, miRNA mimics, miRNA inhibitors and ASOs according to the manufacturer’s instructions. Plasmid transfections in HEK293T and ESCC cells were performed using polyethyleneimine (PEI, Polysciences) and Lipofectamine™3000 reagent (Invitrogen), respectively, according to the manufacturer’s instructions. Lentiviruses were produced by seeding HEK293T cells in culture plates coated with poly-D-lysine (0.1% (w/v), Sigma, P7280) and transfected with lentiviral vectors together with packaging vectors, pMDL, VSVG and REV, at a ratio of 10:5:3:2 using Polyethyleneimine (PEI, Polysciences) for 48 h according to the manufacturer’s instructions. Medium was replaced 24 h later. Viruses were then harvested and filtered. ESCC cells were infected with viruses in the presence of 10 µg/mL polybrene (Sigma, H9268) or stored in − 80 °C. Infected cells were selected with puromycin (Invitrogen) at 1 μg/ml. Targeting sequences of siRNAs and ASOs were listed in Additional file 2: Table S1.

### Competitive endogenous RNA (ceRNA) network analysis

To construct ceRNA network, miRNAs that could bind to LINC00680 were first predicted by using three independent algorithms, miRanda (sequence align score, -sc 150) [40], RNAhybrid (minimal free energy, -e -23) [41], and TarPmiR (probability of target site, -p 0.8) [42], based on miRBase. The miRNAs that were commonly predicted, miR-423-5p, miR-4739, miR-6773-3p, miR-6842-3p, miR-7107-5p, and miR-6791-5p were chosen for downstream analysis. To construct ceRNA network for LINC00680, the mRNA targets that were demonstrated to be positively-regulated by LINC00680 were kept. The ceRNA network was constructed by Cytoscape [43].

### Dual luciferase reporter assay

LINC00680 and the 3’ UTR of PAK6 with the potential miR-423-5p binding sites as well as its mutant forms were designed, synthesized and inserted into pmiR-RB-reporter vectors (RiboBio), which were termed as LINC00680 (WT)-*luc*, PAK6 (WT)-*luc*, LINC00680 (MT)-*luc*, and PAK6 (MT)-*luc*, respectively. Luciferase activity was determined by Dual Luciferase Assay Kit (Promega) in line with the manufacturer’s instructions.

### Cell proliferation and colony formation assays

Cell proliferation was monitored by MTS assay using CellTiter96®Aqueous One Solution Cell Proliferation Assay kit (Promega). Briefly, the transfected ESCC cells were cultured for 24 h and then suspended in 100 μL culture medium containing 10% fetal bovine serum and re-seeded into 96-well plate with 3 × 10^3^ cells per well. After incubation for 0, 24, 48, 72, or 96 h, 20 μL of MTS reagent was added to each well and incubated at 37 °C with 5% CO_2_ for 1 h. The optical density at 490 nm was measured for each sample using a Thermo Multiskan MK3 Microplate Reader.

In the colony formation assay, transfected cells were re-seeded in 6-well plates at a density of 2 × 10^3^ cells per well and cultured for 2 weeks. The cells were then washed twice with PBS, fixed with methanol for 20 min, and stained with 0.1% crystal violet solution for another 20 min at room temperature. Five randomly chosen fields were taken by an inverted microscope to count the number of colonies, and the mean was taken.

### Wound healing and transwell assays

For wound healing assays, the transfected ESCC cells were re-seeded and grown to 90% confluence in 6-well culture plates, and linear scratch wounds were created by a sterile 200 μl pipet tip in the cell monolayer. The wells were washed by PBS for three times to remove detached cells. An inverted microscope (Carl Zeiss) was used to capture the images to calculate the wound area at 0 and 36 h after scratch. The percentage of wound area was calculated as the ratio of scratch area at 36 h compared to that at 0 h in each group.

Cell invasion was investigated by transwell chamber harboring 50 ng/mL Matrigel (Corning) according to manufacturer’s instructions. Briefly, ESCC cells were resuspended in 300 μl serum-free medium and loaded onto the upper chamber at a density of 5 × 10^4^ cells per chamber. Meanwhile, 500 μl medium with 10% FBS was added to underlayer of chamber. After incubation for 24 h, a cotton swab was utilized to wipe off the residual cells on the upper surface of the inner chamber. Meanwhile, invasive cells on the other side of the membrane were fixed with methanol for 20 min and followed by staining with 0.1% crystal violet for 20 min. Finally, five randomly chosen fields were taken by an inverted microscope to count the number of invasive cells, and the mean was taken.

### Western blotting

Cells were lysed with RIPA extraction reagent (Beyotime) supplemented with PMSF protease inhibitor (Beyotime) followed by ultrasound for 30 s and centrifuged at 12,000 rpm for 20 min. The concentration of protein was quantified using the BCA Protein Assay Kit (Beyotime). The same amounts of proteins were separated by 10% SDS-PAGE and transferred onto polyvinylidene fluoride (PVDF) membranes (Millipore). The membrane was blocked using QuickBlock™ (Beyotime) for 1 h at room temperature and incubated with primary antibody at 4 °C overnight. Then the prepared membranes were incubated with horseradish peroxidase-labeled secondary antibody for 2 h at room temperature. The bands on the blots were visualized using an ECL chemiluminescent reagent (Millipore). GAPDH was used as the internal controls. Antibodies against the following proteins were used: PAK6 (A7821, ABclonal), GAPDH (#5174, Cell Signaling Technology). Anti-rabbit IgG (#7074, Cell Signaling Technology) was used as a secondary antibody.

### Subcellular fractionation

Nuclear and cytoplasmic fractions of ESCC cells were prepared by using a PARIS kit protein and RNA isolation system (Life Technologies) according to the manufacturer’s instructions. The amount of LINC00680, actin, and U6 in both fractions were determined by RT-qPCR analysis.

### Xenograft assays in nude mice

Male BALB/c nude mice (4–5 weeks of age, 18–20 g of weight) were used for xenograft models and maintained under SPF conditions in accordance with a protocol approved by the Animal Ethics Committee of Xiamen University.

For xenograft models, KYSE510 cells (3 × 10^5^ in 100 μl sterile PBS) transfected with sh NC, sh LINC00680 #1 and sh LINC00680 #2 were subcutaneously injected in the left flanks of the mice.

For ASO in vivo treatment experiment, KYSE510 cells (3 × 10^5^ in 100 μl sterile PBS) were subcutaneously injected in the left flanks of the mice. Mice were randomized into two groups (*n* = 5 in each group) when the tumor size reached approximately 100 mm^3^. ASOs were delivered by intratumor injection every 5 days at a dose of 5 nmol each mouse (50 mM ASO in 100 μl sterile PBS). Tumors were measured by caliper every 5 days. The tumor volume was calculated as (length × width^2^)/2. Then, mice were euthanized and subcutaneous tumor tissues were dissected, weighed, and photographed at the end of experiments.

Copy number detection.

The exact copy numbers of LINC00680 and miR-423-5p per KYSE510 and KYSE140 cell were quantified by RT-qPCR assay. In this assay, serially diluted RT-PCR products of LINC00680 and miR-423-5p were used as templates to formulate standard curves, and the exact copies of LINC00680 and miR-423-5p per cell were then calculated using the online tool (https://cels.uri.edu/gsc/cndna.html).

### Statistical analysis

**Fig. 1:**
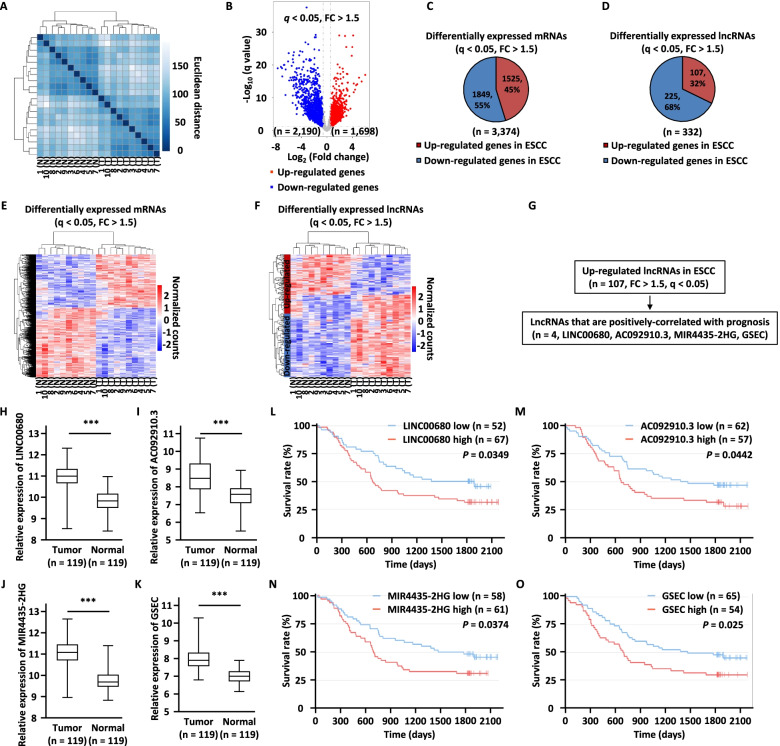
A large number of lncRNAs are dysregulated in ESCC. **a** Ten pairs of ESCC tumor tissues (T) and matched adjacent normal tissues (N) were collected and subjected to RNA-seq analysis followed by hierarchical cluster analysis. **b** Volcano plot shows the fold change against the q value for genes as described in (**a**). Blue and red dots represent genes with significant change (q < 0.05, FC > 1.5). **c-d** Pie chart shows the differentially expressed mRNAs (**c**) and lncRNAs (**d**) in ESCC tumor samples according to RNA-seq. **e–f** Heat map displays the expression of the differentially expressed mRNAs (**e**) and lncRNAs (**f**) as shown in (**c**) and (**d**), respectively. **g** Flowchart to search for clinical-relevant lncRNAs in ESCC. **h–k** The relative expression of LINC00680 (**h**), AC092910.3 (**i**), MIR4435-2HG (**j**), and GSEC (**k**) in ESCC tumor and normal tissues from lnCAR database. **l-o** The correlation between prognosis and the expression of LINC00680 (**l**), AC092910.3 (**m**), MIR4435-2HG (**n**), and GSEC (**o**) in ESCC patients from lnCAR database

Student’s t-tests were used for comparisons between experimental and control conditions, and one-way ANOVA was used for multiple group comparisons. The Chi-square test was used to assess correlations between LINC00680 expression and the clinicopathological features of ESCC patients. Spearman correlation analysis was performed to assess the relationship between different factors. The survival curves were constructed with the Kaplan–Meier method and were performed with the log-rank test for significance. The survival data in Fig. 1l-o were obtained from lnCAR database (https://lncar.renlab.org/). According to the annotation of database, the differential expression analysis was performed by limma package. A robust rank aggregation algorithm was performed to integrate the lncRNA profiles in an unbiased manner. The aggregation rank score (AR score) represents the integrated rank from the meta-analysis of fold change from different microarray studies, in which a larger AR score indicates an up-regulated lncRNA (those lncRNAs distributed in the lncRNA high group), whereas a down-regulated lncRNA (those lncRNAs distributed in the lncRNA low group). The results were expressed as the mean ± the standard deviation (SD) of at least three independent experiments. All statistical analyses were performed using two-tailed *P* values.

## Results

### A large cohort of lncRNAs are differentially expressed in ESCC compared to adjacent normal tissues

In order to identify differentially expressed lncRNAs in ESCC, regular RNA sequencing was performed using ten pairs of ESCC tissues and matched adjacent normal tissues. Hierarchical cluster analysis results indicated that the tumor samples could be well distinguished from the normal ones (Fig. 1a). Differential expression analysis results revealed that 1,698 and 2,190 Refseq genes were up-regulated and down-regulated in tumor samples, respectively (q < 0.05, FC > 1.5) (Fig. 1b). Among which, 1,525 mRNAs and 107 lncRNAs were up-regulated, 1,849 mRNAs and 225 lncRNAs were down-regulated in tumor samples (Fig. 1c, d). The expression patterns of these dysregulated mRNAs (Fig. 1e) and lncRNAs (Fig. 1f) were shown by heat maps. UCSC genome browser views for representative genes that are differentially expressed in normal and ESCC samples were shown (Additional file 1: Figure S1A-S1D). Hallmark gene sets analysis results revealed that genes involved in epithelial mesenchymal transition, interferon gamma response, and E2F targets are the top three most enriched in genes up-regulated in tumors, and genes involved in estrogen response early, fatty acid metabolism, and xenobiotic metabolism are the top three most enriched in genes down-regulated in tumors, which are in consistent with our recent proteomic analysis (Additional file 1: Figure S1E, S1F) [44].

We focused on studying lncRNAs which were found upregulated in ESCC in the current study. LncRNAs, such as LUCAT1, KCNMB2-AS1, CASC15, MIAT, PTOV1-AS2, LINC00680, and RNF217-AS1 [45–50] were reported to be up-regulated in ESCC and other malignant tumors, validating our RNA-sequencing results. In particular, the high expression of four lncRNAs including LINC00680, AC092910.3, MIR4435-2HG, and GSEC were found to be correlated with poor prognosis in ESCC patients in lnCAR database [51], suggesting they are functionally important and clinically relevant (Fig. 1g-o and Additional file 3: Table S2).

### LINC00680 is highly expressed in ESCC tissues and required for ESCC cell proliferation, colony formation, migration, and invasion

**Fig. 2:**
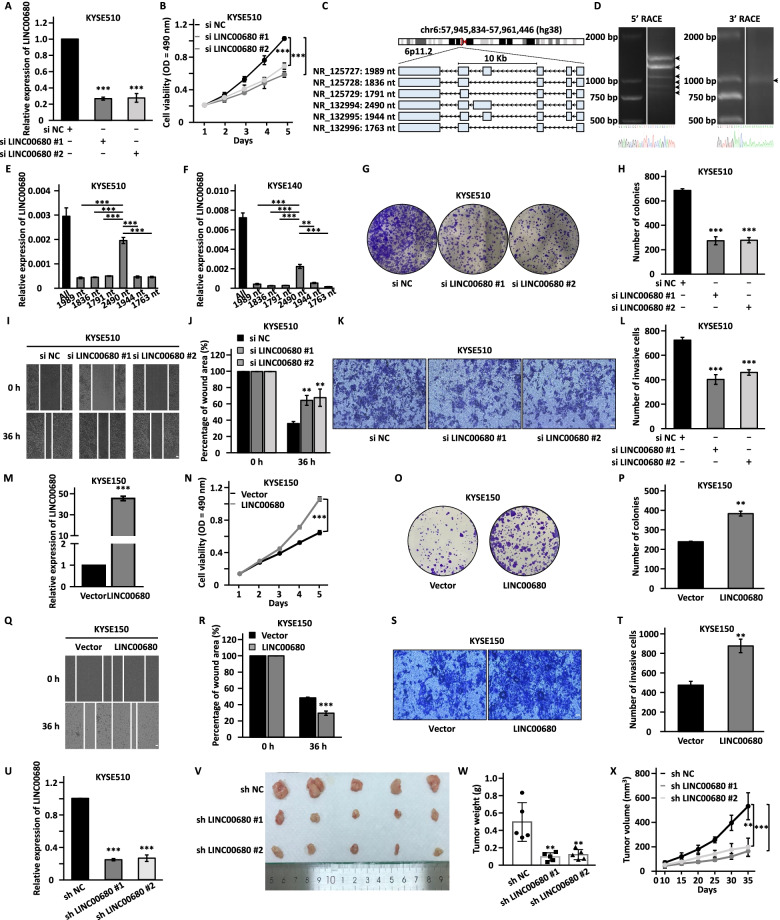
LINC00680 promotes cell proliferation, colony formation, migration, and invasion in vitro and tumor growth in vivo. **a, b, g, i, k** KYSE510 cells were transfected with negative control siRNA (si NC) or two independent siRNAs targeting LINC00680 (si LINC00680 #1 and si LINC00680 #2) followed by RT-qPCR analysis (**a**), cell proliferation assay (**b**), colony formation assay (**g**), wound healing assay (**i**), and transwell assay (**k**). Scale bar: 50 µm. **h, j, l** Quantification of the number of colonies (**h**), the percentage of wound area (**j**), and the number of invasive cells (**l**) as shown in (**g**), (**i**), and (**k**), respectively. **c** Schematic diagram of the chromosomal location of LINC00680 and the six isoforms confirmed by RACE and PCR assays. **d** cDNA from KYSE140 cells were subjected to 5’ and 3’ RACE assays to detect the full sequence of LINC00680. PCR products were separated by DNA agarose gel. The six isoforms were indicated by arrows. DNA marker was shown on the left. Sanger sequencing results of the 5’ and 3’ end from PCR products were shown at the bottom, detailed sequencing results could be found in Additional file 4: Table S3. **m, n, o, q, s** KYSE150 cells were transfected with control vector or vector expressing LINC00680 followed by RT-qPCR analysis (**m**), cell proliferation assay (**n**), colony formation assay (**o**), wound healing assay (**q**), and transwell assay (**s**). Scale bar: 50 µm. **p, r, t** Quantification of the number of colonies (**p**), the percentage of wound area (**r**), and the number of invasive cells (**t**) as shown in (**o**), (**q**), and (**s**), respectively. **u** KYSE510 cells stably transfected with negative control shRNA (sh NC) or two independent shRNAs targeting LINC00680 (sh LINC00680 #1 and sh LINC00680 #2) were subjected to RT-qPCR analysis. **v** Cells as described in (**u**) were subcutaneously injected into nude mice (*n* = 5 in each group), and images of excised tumors were shown. **w** The average of the weight of tumors as shown in (**v**). **x** The growth curves of tumors as shown in (**v**). The experiments were repeated for three times, and representative data is shown with mean ± standard deviation (SD), *P < 0.05, **P < 0.01, ***P < 0.001

To test whether the four lncRNAs identified above are functional important, we transfected KYSE510 cells with two independent siRNAs targeting each individual lncRNA followed by cell proliferation assay. Notably, knockdown of LINC00680 had the most dramatic effects on cell proliferation (Fig. 2a, b and Additional file 1: Figure S2A, S2B). We therefore focused on investigating the functions and molecular mechanisms of LINC00680 in the current study. To confirm the full-length of LINC00680, 5’ and 3’ rapid amplification of the cDNA ends (RACE) and PCR assays were performed. Six different isoforms with 1989 nucleotides (nt), 1836 nt, 1791nt, 2490 nt, 1944 nt, and 1763 nt in length were identified, which share the same transcriptional start site and end site (Fig. 2c, d and Additional file 4: Table S3). The expression of the 2490 nt isoform was significantly higher than the other ones (Fig. 2e, f). To further examine the functional importance of LINC00680 in ESCC, we transfected KYSE510 cells with two independent siRNAs targeting LINC00680 followed by colony formation, wound healing, and transwell assay. LINC00680 knockdown mitigated colony formation, migration, and invasion ability (Fig. 2g-l). The requirement of LINC00680 in cell proliferation, colony formation, migration, and invasion in ESCC cells was also demonstrated in another ESCC cell line, KYSE140 (Additional file 1: Figure S2C-S2J). LINC00680 overexpression (the 2490-nt isoform) was also performed in KYSE150 cells, which has relatively low expression of LINC00680 (Additional file 1: Figure S2K). As expected, overexpression of LINC00680 promoted cell proliferation, colony formation, cell migration, and invasion in KYSE150 cells (Fig. 2m-t). Then, to explore the effects of LINC00680 on tumor growth in vivo, equal amounts of KYSE510 cells stably transfected with control shRNA or two independent shRNAs targeting LINC00680 were inoculated into BALB/c nude mice (Fig. 2u). Tumor growth rate and size were dramatically reduced when LINC00680 was knocked down (Fig. 2v-x). To strengthen the significance, LINC00680 was found to present at a much higher level in ESCC tissues than adjacent normal tissues (Additional file 1: Figure S2L), and high expression of LINC00680 was positively associated with poor prognosis in ESCC patients (Additional file 1: Figure S2M) in our in-house cohort. In addition, high expression of LINC00680 was found to be correlated with tumor size, depth of invasion, lymph node metastasis and TNM stage (Additional file 5: Table S4). Taken together, LINC00680 is highly expressed in ESCC and participates in ESCC tumorigenesis.

### LINC00680 is localized in the cytosol of cells and regulates the expression of PAK6

**Fig. 3:**
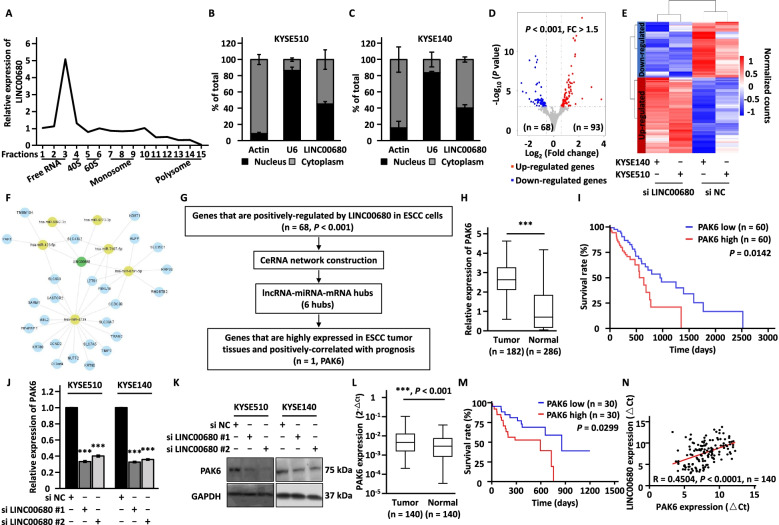
Transcriptomic analysis reveals that PAK6 is regulated by LINC00680. **a** KYSE510 cells were subjected to polysome profiling and the resultant fractions were applied to RNA exaction and RT-qPCR analysis to examine the expression of LINC00680. Fractions 1 to 3: Free RNA (unbound with ribosome); Fraction 4: 40S (40S ribosomal subunit); Fractions 5 and 6: 60S (60S ribosomal subunit); Fractions 7 to 9: Monosome; Fractions 10 to 15: Polysome. **b-c** The subcellular distribution of LINC00680 in KYSE510 (**b**) and KYSE140 (**c**) cells was determined by subcellular fractionation followed by RT-qPCR analysis. **d** KYSE510 and KYSE140 cells transfected with negative control siRNA (si NC) and siRNA specifically targeting LINC00680 (si LINC00680) for three days were subjected to RNA-seq analysis, and gene expression patterns are presented by volcano plot. Blue and red dots represent genes with significant change (*P* < 0.001, FC > 1.5). **e** The expression of differentially expressed genes (*P* < 0.001, FC > 1.5) was represented by heat map. **f** CeRNA network constituting of LINC00680-miRNAs-mRNAs (genes positively-regulated by LINC00680, *n* = 68) was shown. Nodes in green, yellow, and light blue represent LINC00680, miRNAs, and mRNAs, respectively. **g** The flowchart to identify clinically relevant target genes of LINC00680 in ESCC. **h** The expression of PAK6 in a cohort of esophageal carcinoma (ESCA) samples (*n* = 182) and normal samples (*n* = 286) from GEPIA database (http://gepia.cancer-pku.cn/). **i** The correlation between the expression of PAK6 and prognosis of ESCA patients from Oncolnc database (http://www.oncolnc.org/). **j-k** KYSE510 and KYSE140 cells were transfected with negative control siRNA (si NC) or two independent siRNAs targeting LINC00680 (si LINC00680 #1 and si LINC00680 #2) followed by RT-qPCR (**j**) and immunoblotting analysis (**k**). **l** The expression of PAK6 in a cohort of ESCC tumor samples (*n* = 140) and normal samples (*n* = 140) in house. **m** The correlation between the expression of PAK6 and prognosis of ESCC patients in house (n = 60). **n** The correlation between the expression of PAK6 and LINC00680 in ESCC tumor samples in house (*n* = 140). The experiments were repeated for three times, and representative data is shown with mean ± standard deviation (SD), *P < 0.05, **P < 0.01, ***P < 0.001

To understand the molecular mechanisms underlying LINC00680 regulation of ESCC tumorigenesis, we first sought to examine whether LINC00680 has coding potential by performing polysome profiling. The results showed that LINC00680 was largely associated with ribosome-free fractions, indicating that it was truly a non-coding RNA (Fig. 3a), which were in line with the results from CPAT, a web tool for predicting coding potential (http://lilab.research.bcm.edu/) [52] (Additional file 1: Figure S3A). The regulatory modes of lncRNAs are largely associated with its subcellular localization. We therefore assessed the cellular localization of LINC00680 by performing cellular fractionation followed by RNA extraction and RT-qPCR analysis. It was determined that LINC00680 was existed both in the cytoplasm and nucleus of ESCC cells (Fig. 3b, c). To gain insights into the molecular mechanisms through which LINC00680 regulates ESCC, transcriptome analysis was performed in KYSE510 and KYSE140 cells transfected with control siRNA or siRNA specifically targeting LINC00680 to identify target genes regulated by LINC00680. Differential expression analysis results revealed that there were 68 and 93 genes positively and negatively-regulated by LINC00680, respectively, in both cell lines (FC > 1.5, *P* < 0.001) (Fig. 3d, e). As LINC00680 is largely localized in the cytoplasm, we explore the possibility that LINC00680 functions as a ceRNA to sponge miRNA to regulate its target genes [9, 53–55]. To this end, three different algorithms (TarPmiR, miRanda, and RNAhybrid) were utilized to predict potential miRNAs that can bind to LINC00680 at high stringency, and then the highly confident miRNAs predicted were overlapped (Additional file 1: Figure S3B). Furthermore, lncRNA (LINC00680)-miRNA-mRNA (genes positively-regulated by LINC00680) network was constructed to connect LINC00680 with its target genes with miRNAs. As depicted, six miRNAs including miR-423-5p, miR-6842-3p, miR-6773-3p, miR-7107-5p, miR-6791-5p, and miR-4739, as well as twenty-four target genes including PAK6, SLC43A2, ALPP, KRT80, LZTS1 et al. were involved in the ceRNA network (Fig. 3f). We then searched for target genes that are clinically relevant (i.e. highly expressing in ESCC tumor tissues and positively correlated with poor prognosis of ESCC patients) as LINC00680, which led to the discovery of one gene named PAK6 (Fig. 3g-i and Additional file 6: Table S5). The effect of knockdown of LINC00680 on PAK6 expression at mRNA and protein level was explored using RT-qPCR and western blot analysis, respectively (Fig. 3j, k). The high expression of PAK6 in ESCC tissues and the correlation with poor prognosis was independently confirmed in our in-house cohort (Fig. 3l, m). Importantly, the expression of PAK6 was positively correlated with that of LINC00680 (Fig. 3n). Collectively, these results indicated that PAK6 might be a downstream target gene of LINC00680, with implications in ESCC.

### PAK6 is a critical downstream target of LINC00680 to promote ESCC cell proliferation, colony formation, migration, and invasion

**Fig. 4:**
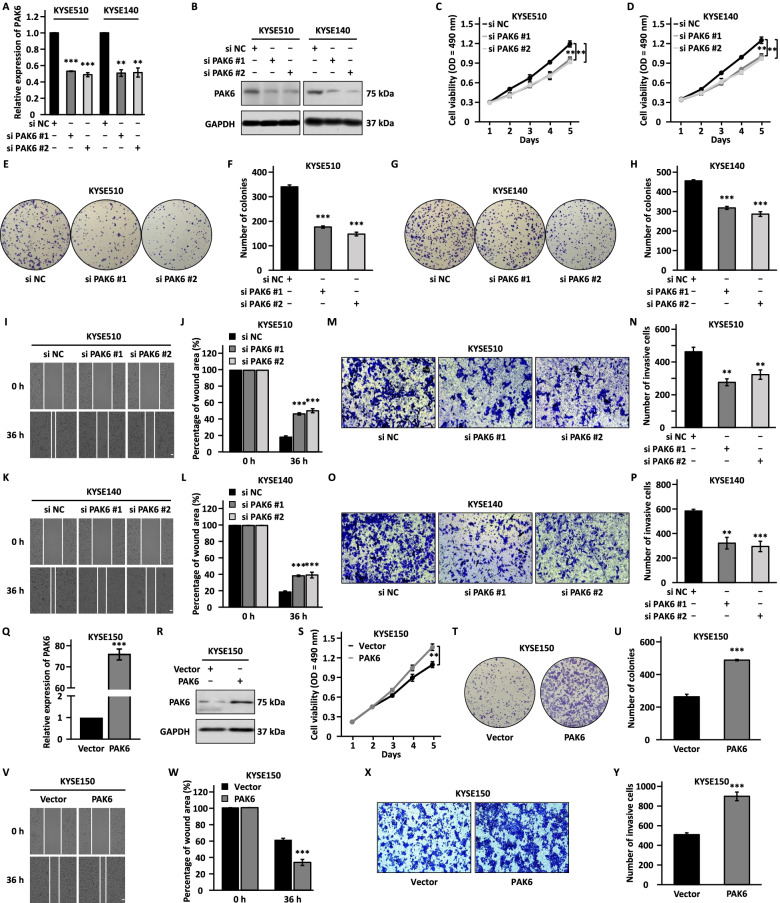
PAK6 promotes the malignant behaviors in ESCC cells. **a, b, c, d, e, g, i, k, m, o** KYSE510 and KYSE140 cells were transfected with negative control siRNA (si NC) or siRNA targeting PAK6 (si PAK6 #1 and si PAK6 #2) followed by RT-qPCR analysis (**a**), immunoblotting analysis (**b**), cell proliferation assay (**c-d**), colony formation assay (**e, g**), wound healing assay (**i, k**), and transwell assay (**m, o**). Scale bar: 50 µm. **f, h, j, l, n, p** Quantification of the number of colonies (**f, h**), the percentage of wound area (**j, l**), and the number of invasive cells (**n, p**). **q, r, s, t, v, x** KYSE150 cells were transfected with control vector or vector expressing PAK6 followed by RT-qPCR analysis (**q**), immunoblotting analysis (**r**), cell proliferation assay (**s**), colony formation assay (**t**), wound healing assay (**v**), and transwell assay (**x**). Scale bar: 50 µm. **u, w, y** Quantification of the number of colonies (**u**), the percentage of wound area (**w**), and the number of invasive cells (**y**) as shown in (**t**), (**v**), and (**x**), respectively. The experiments were repeated for three times, and representative data is shown with mean ± standard deviation (SD), *P < 0.05, **P < 0.01, ***P < 0.001

To examine whether PAK6 is truly a functional target for LINC00680, we first asked whether PAK6 is involved in the malignant behaviors of ESCC cells. KYSE510 and KYSE140 cells were transfected with control siRNA or two independent siRNAs specifically targeting PAK6 followed by cell proliferation, colony formation, wound healing, and transwell assays. The knockdown efficiency was confirmed by RT-qPCR and western blot in both ESCC cell lines (Fig. 4a, b). Knockdown of PAK6 resulted in significant suppression of cell proliferation, colony formation, migration, and invasion (Fig. 4c-p). To further support the functional importance of PAK6, PAK6 overexpression exhibited the opposite effects, promoting cell proliferation, colony formation, migration, and invasion (Fig. 4q-y).

**Fig. 5:**
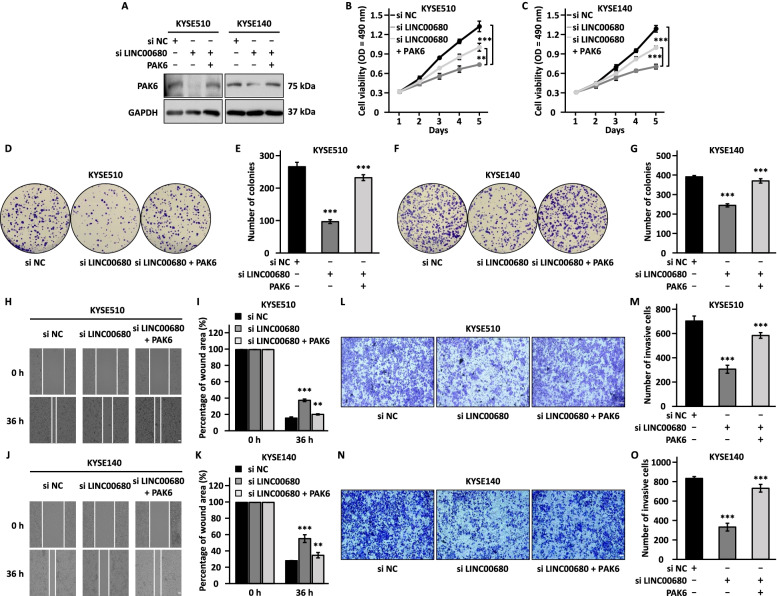
PAK6 restoration attenuates the inhibitory effects of LINC00680 knockdown on the malignant behaviors in ESCC cells. **a, b, c, d, f, h, j, l, n** KYSE510 and KYSE140 cells were transfected with negative control siRNA (si NC) or siRNA targeting LINC00680 (si LINC00680) in the presence or absence of vector expressing PAK6 followed by immunoblotting analysis (**a**), cell proliferation assay (**b-c**), colony formation assay (**d, f**), wound healing assay (**h, j**), and transwell assay (**l, n**). Scale bar: 50 µm. **e, g, j, k, m, o** Quantification of the number of colonies (**e, g**), the percentage of wound area (**i, k**), and the number of invasive cells (**m, o**). The experiments were repeated for three times, and representative data is shown with mean ± standard deviation (SD), *P < 0.05, **P < 0.01, ***P < 0.001

We next tested whether PAK6 is a functional downstream target of LINC00680 by performing rescue experiments, in which PAK6-overexpression vector was transfected when LINC00680 was knocked down in KYSE510 and KYSE140 cells. It was revealed that the expression of PAK6 was decreased by LINC00680 knockdown, which was rescued when PAK6 was overexpressed (Fig. 5a). Results from cell proliferation, colony formation, wound healing, and transwell assays revealed that the defects caused by LINC00680 knockdown were effectively alleviated by PAK6 overexpression (Fig. 5b-o). Overall, PAK6 is a functional downstream target gene of LINC00680 to promote ESCC malignant behaviors.

### LINC00680 serves as a miRNA sponge for miR-423-5p to regulate the expression of PAK6 and promote ESCC cell proliferation, colony formation, migration, and invasion

**Fig. 6:**
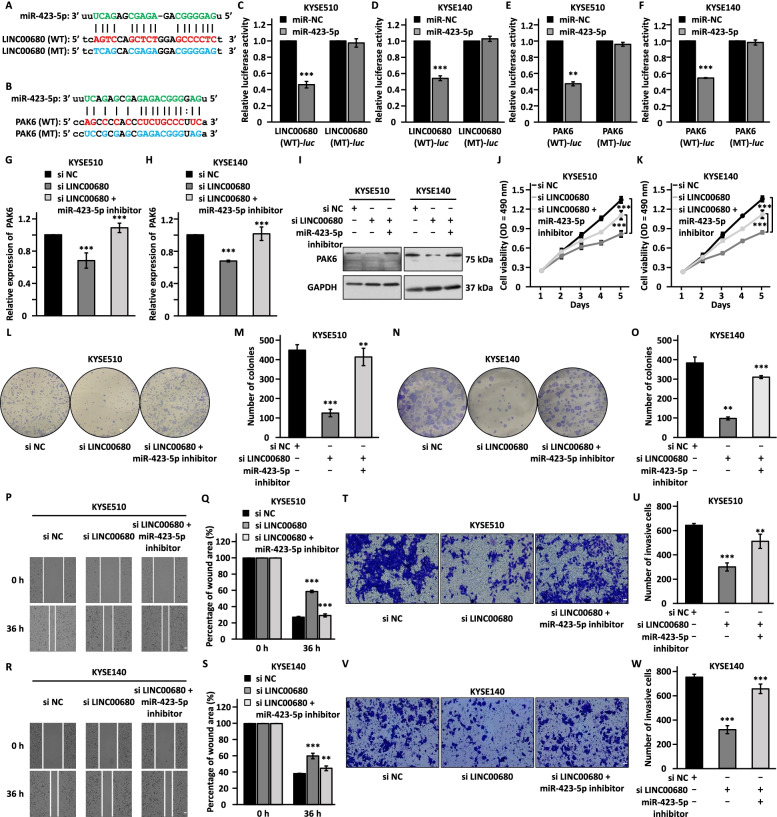
LINC00680 acts as a miRNA sponge for miR-423-5p to regulate the expression of PAK6 and the malignant behaviors in ESCC cells**. a-b** Sequence match between miR-423-5p and wild-type (WT) LINC00680 (**a**) or the 3’ UTR of PAK6 (**b**) as well as the corresponding mutant form (MT) with the predicted miR-423-5p binding site mutated is shown. **c-d** KYSE510 (**c**) and KYSE140 (**d**) cells were transfected with luciferase reporter vectors containing wild-type (WT-*luc*) or mutated (MT-*luc*) LINC00680 in the presence or absence of negative control miRNA (miR-NC) or miR-423-5p mimic (miR-423-5p) followed by dual-luciferase reporter assay. **e–f** KYSE510 (**e**) and KYSE140 (**f**) cells were transfected with luciferase reporter vectors containing wild-type (WT-*luc*) or mutated (MT-*luc*) 3’ UTR of PAK6 in the presence or absence of negative control miRNA (miR-NC) or miR-423-5p mimic (miR-423-5p) followed by dual-luciferase reporter assay. **g, h, i, j, k, l, n, p, r, t, v** KYSE510 and KYSE140 cells were transfected with negative control siRNA (si NC) or siRNA targeting LINC00680 (si LINC00680) in the presence or absence of miR-423-5p inhibitor followed by RT-qPCR analysis (**g, h**), immunoblotting analysis (**i**), cell proliferation assay (**j, k**), colony formation assay (**l, n**), wound healing assay (**p, r**), and transwell assay (**t, v**). Scale bar: 50 µm. **m, o, q, s, u, w** Quantification of the number of colonies (**m, o**), the percentage of wound area (**q, s**), and the number of invasive cells (**u, w**). The experiments were repeated for three times, and representative data is shown with mean ± standard deviation (SD), *P < 0.05, **P < 0.01, ***P < 0.001

As revealed by the ceRNA network, LINC00680 could regulate PAK6 expression via sponging miR-423-5p (Fig. 3f). miR-423-5p was reported to be involved in many human cancers, and it was found to be downregulated in ESCC tissue samples (Additional file 1: Figure S4A) [56–58]. We first examined whether miR-423-5p can bind to LINC00680 and the 3’ UTR of PAK6. The starBase website was used to predict the potential miRNA binding sites. The consequential pairing of target regions of LINC00680 and the 3’ UTR of PAK6 with the highest targets score were shown (Fig. 6a, b). Luciferase reporter vectors containing the binding regions for miR-423-5p, either wild-type (WT-*luc*) or mutated form (MT-*luc*), were constructed, which were transfected into KYSE510 and KYSE140 cells with control miRNA (miR-NC) or miR-423-5p mimic (miR-423-5p) followed by dual-luciferase assay. The results showed that co-transfection of miR-423-5p significantly reduced the luciferase activity of LINC00680 (WT)-*luc* and PAK6 (WT)-*luc* reporters compared with the miR-NC group, whereas there was no significant change for LINC00680 (MT)-*luc* or PAK6 (MT)-*luc* (Fig. 6c-f). To support that LINC00680 could serve as a sponge for miR-423-5p, the copy number of LINC00680 and miR-423-5p was approximately 241 and 136 copies per cell, respectively, in KYSE510 cells (Additional file 1: Figure S4B, S4C and S4D left panel). Similar results were obtained in KYSE140 cells, with that 210 and 178 copies were detected for LINC00680 and miR-423-5p, respectively (Additional file 1: Figure S4D right panel). Likewise, the copy number of LINC00680 was significantly higher than that of miR-423-5p in ESCC tissue samples (Additional file 1: Figure S4E).

We next asked whether LINC00680 regulates the expression of PAK6 and exerts its oncogenic role is dependent on miR-423-5p. KYSE150 cells were infected with a control vector or vectors expression wild-type LINC00680 (WT) or LINC00680 with the miR-423-5p mutated (MT) followed by RT-qPCR and immunoblotting analysis to examine the mRNA and protein levels of PAK6, respectively. The results showed that PAK6, from both mRNA and protein level, was significant upregulated when LINC00680 (WT), but not LINC00680 (MT), was introduced (Additional file 1: Figure S4F-S4H). Furthermore, LINC00680 (WT), but not LINC00680 (MT), promoted KYSE150 cell proliferation, colony formation, migration, and invasion (Additional file 1: Figure S4I-S4O). To strengthen that LINC00680 regulates the expression of PAK6 and exerts its oncogenic role is dependent on miR-423-5p, the expression of PAK6, both at mRNA and protein level, was declined by LINC00680 knockdown, which was obviously reversed in the presence of miR-423-5p inhibitor in both KYSE510 and KYSE140 cells (Fig. 6g-i). Furthermore, inhibition of miR-423-5p partially abrogated the defects in cell proliferation, colony formation, migration, and invasion caused by LINC00680 knockdown in both KYSE510 and KYSE140 cells (Fig. 6j-w). Collectively, our data supported that LINC00680 regulates the expression of PAK6 and promotes malignant phenotypes in ESCC through sponging miR-423-5p.

### LINC00680 is a potential therapeutic target for ESCC

**Fig. 7:**
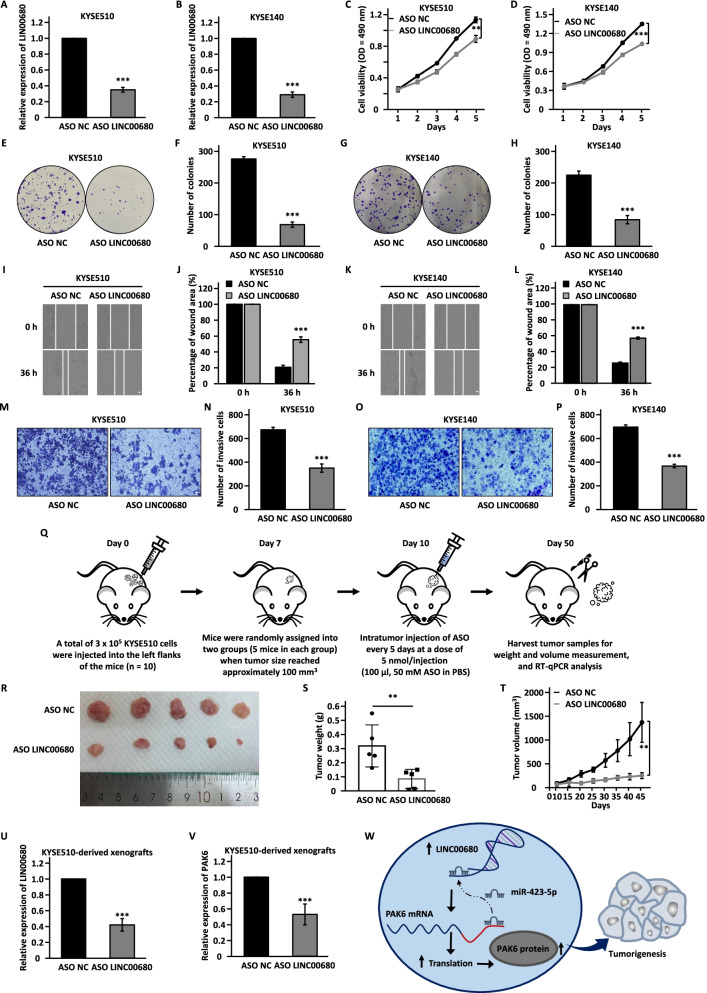
LINC00680 is a potential therapeutic target in ESCC. **a, b, c, d, e, g, i, k, m, o** KYSE510 and KYSE140 cells were transfected with negative control ASO (ASO NC) or ASO specifically targeting LINC00680 (ASO LINC00680) followed by RT-qPCR analysis (**a, b**), cell proliferation assay (**c, d**), colony formation assay (**e, g**), wound healing assay (**i, k**), and transwell assay (**m, o**). Scale bar: 50 µm. **f, h, j, l, n, p** Quantification of the number of colonies (**f, h**), the percentage of wound area (**j, l**), and the number of invasive cells (**n, p**). **q** Graphic illustration of ASO NC or ASO LINC00680 injection in nude mice. **r** Images of excised tumors as described in (**q**) are shown. **s** The average of the weight of tumors as shown in (**r**). (**t**) The growth curves of tumors as shown in (**r**). **u, v** The expression of LINC00680 (**u**) and PAK6 (**v**) in tumors as described in (**r**) was examined by RT-qPCR analysis. **w** A proposed model of LINC00680 function in ESCC. The highly expressed LINC00680 in ESCC cells functions as a miRNA sponge to sponge miR-423-5p to release its repression on PAK6, leading to the aberrant expression of PAK6 and ESCC tumorigenesis. The experiments were repeated for three times, and representative data is shown with mean ± standard deviation (SD), *P < 0.05, **P < 0.01, ***P < 0.001

Currently, anti-sense oligonucleotide (ASO) gained increasing attention owing to their ability to specifically target and degrade target RNA, which has been validated both in vitro and in vivo [32–35, 59, 60]. The upregulation of LINC00680 in ESCC tumor samples and its significant contribution to ESCC malignant phenotypes prompted us to exploit the potential of LINC00680 as a therapeutic target by using ASO. Therefore, ASO specifically targeting LINC00680 (ASO LINC00680) and negative control (ASO NC) were designed and transfected into KYSE510 and KYSE140 cells followed by cell proliferation, colony formation, wound healing, and transwell assays. The knockdown efficiency of ASO was examined by RT-qPCR analysis (Fig. 7a, b). It was demonstrated that cell proliferation, colony formation, migration, and invasion were significantly impaired upon LINC00680 interference by ASO compared with negative control group (Fig. 7c-p). To assess the in vivo anti-growth efficacy of ASO LINC00680, mice were inoculated with KYSE510 cells, randomly assigned into two groups, and then treated with ASO NC or ASO LINC00680 by intratumor injection every 5 days (Fig. 7q). Compared to ASO NC-treated group, tumor growth was significantly suppressed in ASO LINC00680-treated group (Fig. 7r-t). Moreover, the expression of LINC00680 and PAK6 were significantly decreased in tumors treated with ASO LINC00680 (Fig. 7u, v). In conclusion, the current data suggested that LINC00680 might serve as a therapeutic target in ESCC, and ASO targeting LINC00680 represents a promising avenue for the treatment of ESCC patients.

## Discussion

A growing body of studies demonstrated that dysregulated lncRNAs may play essential roles in the occurrence and progression of various diseases, including cancers [17–20, 61, 62]. Accumulated researches have focused on the functions and mechanisms of lncRNAs to discover novel diagnostic markers and therapeutic targets for cancers [63–66]. In this study, a large number of lncRNAs were identified to be expressed aberrantly through transcriptomic analysis of ESCC tumor samples and matched normal tissues, of which LUCAT1 [45], CASC15 [47], MIAT [48], ZNF503-AS1, [67] and STXBP5-AS1 [68] have been shown to either promote or inhibit tumorigenesis in ESCC as well as other malignant tumors. In particular, the high expression of LINC00680, AC092910.3, MIR4435-2HG, and GSEC were found to be correlated with poor prognosis in ESCC patients. Thus, the biological functions and mechanisms of these lncRNAs in ESCC are worth to be investigated.

Results from cell proliferation assay revealed that knockdown of LINC00680 had the most dramatic effects on cell proliferation. Previous studies have demonstrated the oncogenic role of LINC00680 in lung cancer [21, 22], hepatocellular carcinoma [23] and glioblastoma [24], but the functions, molecular mechanisms, and clinical relevance of LINC00680 in ESCC remains largely unknown. In the present study, we demonstrated that LINC00680 acts as an oncogenic lncRNA, which is significantly enriched in ESCC tumor tissues compared to the adjacent normal epithelial tissues, related with tumor size, depth of invasion, lymph node metastasis, and TNM stage, and predicted worse clinical outcomes. Gain- and loss-of-function studies indicated that LINC00680 exerts an oncogenic role in ESCC both in vitro and in vivo.

PAK6, a serine-threonine kinase belonging to the class II p21-activated kinase (PAK) family, is generally overexpressed or hyperactivated in multiple human cancers [69–72], and acts as an oncogene by promoting a number of cancer hallmarks including cancer initiation, cell growth, EMT, and metastasis [73, 74]. In our study, PAK6 was found to be regulated by LINC00680 through RNA-seq analysis; PAK6 was highly expressed in ESCC tumor samples; a positive relationship was revealed between the expression of LINC00680 and PAK6 in ESCC tumor samples. All these strongly indicated that PAK6 is a downstream target of LINC00680 in ESCC. Indeed, PAK6 knockdown clearly inhibited cell proliferation, colony formation, migration, and invasion in ESCC. Functional rescue experiments confirmed that PAK6 restoration reversed the antitumor performance induced by LINC00680 knockdown. The regulatory mechanisms underlying PAK6 in ESCC remain undefined and worth to be investigated.

LncRNAs can sponge miRNAs to relieve the repression of target mRNAs at a post-transcriptional level and subsequently modulate tumor development [27, 75]. In our study, a large portion of LINC00680 was found to be distributed in the cytoplasm of ESCC cells. Consequently, ceRNA network combined with bioinformatics methods predicted that miR-423-5p is a bridge connecting PAK6 and LINC00680. To date, miR-423-5p had been proved to be involved in multiple malignant behaviors by acting as a tumor suppressor or promoter in previous studies [56–58, 76]. Out data indicated that miR-423-5p could bind to both LINC00680 and PAK6, and LINC00680 knockdown led to decreased expression of PAK6, which could be partially reversed by miR-423-5p inhibition. We concluded that LINC00680 regulates PAK6 expression by competitively binding to miR-423-5p, and accelerates tumorigenesis of ESCC cells. It should also be noted that LINC00680 can also sponge miR-568 to upregulate the expression of AKT3 in hepatocellular carcinoma to enhance carcinogenetic stemness behavior and chemoresistance [23]. Therefore, the potential of LINC00680 as a prognostic biomarker and therapeutic target in lncRNA-based cancer therapy warrants further exploration.

ASOs have been used to target mRNAs in vivo, and several ASOs have been investigated in the clinical trials [59, 60]. Discovery of various ASO-based therapeutics and their vital roles in cancer initiation, migration, and metastasis has opened up new horizons in cancer research [28, 31, 77–79]. Compared with traditional RNA interference (RNAi) technologies such as siRNA, ASOs have certain advantages for clinical practice, such as longer half-life, higher cellular uptake efficacy, and stronger silencing effects [80, 81]. In addition, combination therapy with ASOs and other currently approved anti-cancer drugs could be an effective way in cancer therapy. Here, we found that ASO targeting LINC00680 substantially restrained cell proliferative, migratory, and invasive property in vitro and tumor growth ability in vivo. The evidence observed above supports that ASO targeting LIN00680 may serve as a promising therapeutic approach to retard LINC00680-promoted ESCC carcinogenesis.

To conclude, our study demonstrated that LINC00680 acts as an oncogenic lncRNA to promote ESCC, and is correlated with poor prognosis in ESCC. LINC00680/miR-423-5p/PAK6 axis may serve as promising diagnostic and prognostic biomarkers, and therapeutic targets for ESCC patients (Fig. 7w).

## Supplementary Information

**Additional file 1:** **Figure S1.** **Figure S2.** **Figure S3.** **Figure S4.**

**Additional file 2:** **TableS1.**

**Additional file 3:** **TableS2.**

**Additional file 4:** **TableS3.**

**Additional file 5:** **TableS4.**

**Additional file 6:** **TableS5.**

## Data Availability

All the data supporting the findings of this study are available within the article and its additional files and from the corresponding author upon reasonable request.

## Abbreviations

ESCC: Esophageal squamous cell carcinoma
lncRNA: Long non-coding RNA
mRNA: Messenger RNA
PAK6: P21-activated kinase 6
RNA-seq: RNA-sequencing
ceRNA: Competitive endogenous RNA
miRNA: MicroRNA
ASO: Antisense oligonucleotides
RT-qPCR: Real-time quantitative polymerase chain reaction
siRNA: Small interfering RNA
shRNA: Short hairpin RNA
FC: Fold change
