# Supplementary material for: Long non-coding RNA LINC00680 functions as a ceRNA to promote esophageal squamous cell carcinoma progression through the miR-423-5p/PAK6 axis
Source: Mol Cancer. 2022 Mar 7;21:69. doi: 10.1186/s12943-022-01539-3 (PMC8900330; doi:10.1186/s12943-022-01539-3)
Supplement: Supplementary file 1 — Additional file 1: Figure S1. Figure S2. Figure S3. Figure S4. [file 12943_2022_1539_MOESM1_ESM.pdf]

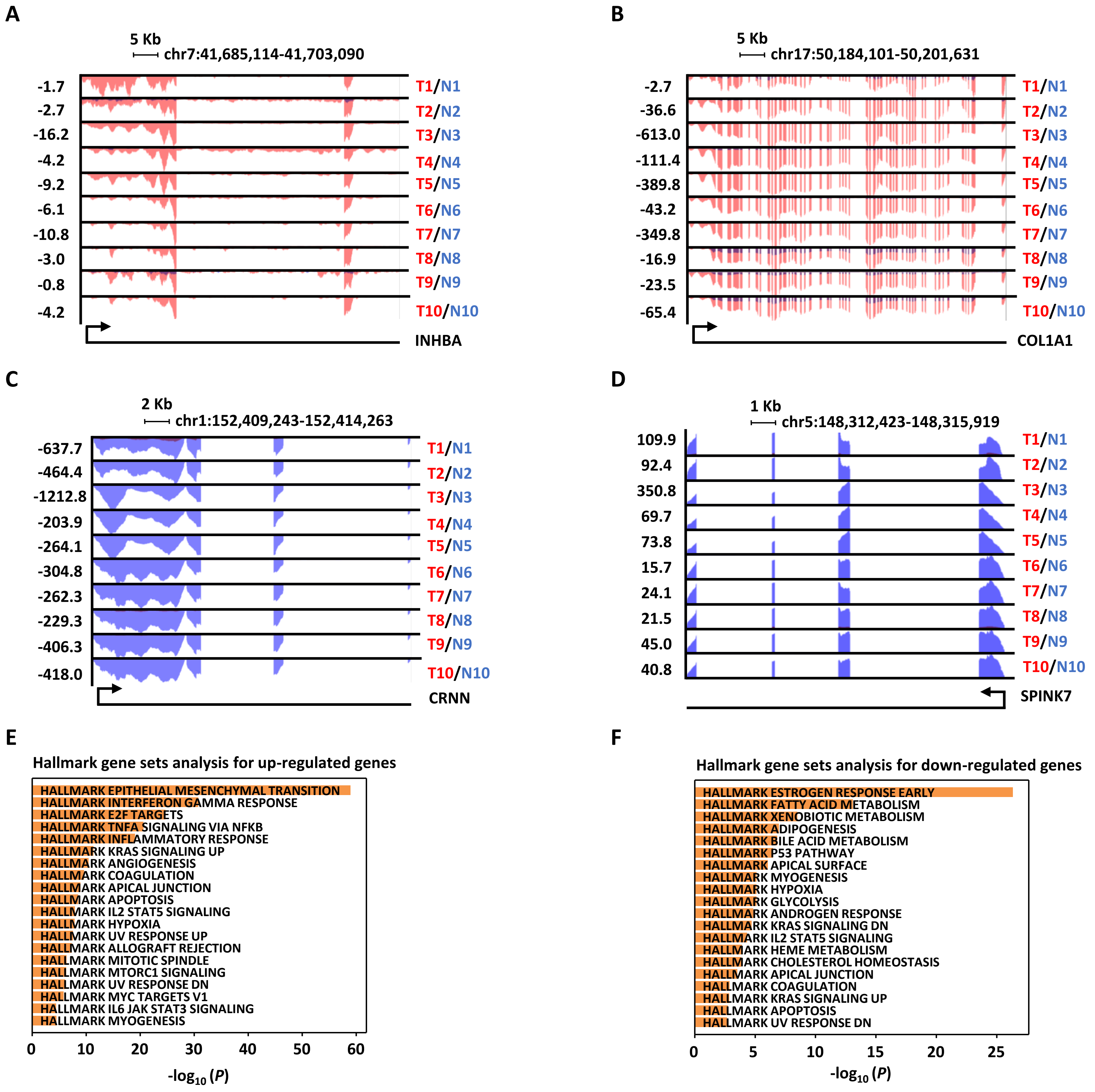
A
5 Kb
chr7:41,685,114-41,703,090
-1.7
-2.7
-16.2
-4.2
-9.2
-6.1
-10.8
-3.0
-0.8
-4.2
T1/N1
T2/N2
T3/N3
T4/N4
T5/N5
T6/N6
T7/N7
T8/N8
T9/N9
T10/N10
INHBA
B
5 Kb
chr17:50,184,101-50,201,631
-2.7
-36.6
-613.0
-111.4
-389.8
-43.2
-349.8
-16.9
-23.5
-65.4
T1/N1
T2/N2
T3/N3
T4/N4
T5/N5
T6/N6
T7/N7
T8/N8
T9/N9
T10/N10
COL1A1
C
2 Kb
chr1:152,409,243-152,414,263
-637.7
-464.4
-1212.8
-203.9
-264.1
-304.8
-262.3
-229.3
-406.3
-418.0
T1/N1
T2/N2
T3/N3
T4/N4
T5/N5
T6/N6
T7/N7
T8/N8
T9/N9
T10/N10
CRNN
D
1 Kb
chr5:148,312,423-148,315,919
109.9
92.4
350.8
69.7
73.8
15.7
24.1
21.5
45.0
40.8
T1/N1
T2/N2
T3/N3
T4/N4
T5/N5
T6/N6
T7/N7
T8/N8
T9/N9
T10/N10
SPINK7
E
Hallmark gene sets analysis for up-regulated genes
HALLMARK EPITHELIAL MESENCHYMAL TRANSITION
HALLMARK INTERFERON GAMMA RESPONSE
HALLMARK E2F TARGETS
HALLMARK TNFA SIGNALING VIA NFKB
HALLMARK INFLAMMATORY RESPONSE
HALLMARK KRAS SIGNALING UP
HALLMARK ANGIOGENESIS
HALLMARK COAGULATION
HALLMARK APICAL JUNCTION
HALLMARK APOPTOSIS
HALLMARK IL2 STAT5 SIGNALING
HALLMARK HYPOXIA
HALLMARK UV RESPONSE UP
HALLMARK ALLOGRAFT REJECTION
HALLMARK MITOTIC SPINDLE
HALLMARK MTORC1 SIGNALING
HALLMARK UV RESPONSE DN
HALLMARK MYC TARGETS V1
HALLMARK IL6 JAK STAT3 SIGNALING
HALLMARK MYOGENESIS
0 10 20 30 40 50 60
-log10 (P)
F
Hallmark gene sets analysis for down-regulated genes
HALLMARK ESTROGEN RESPONSE EARLY
HALLMARK FATTY ACID METABOLISM
HALLMARK XENOBIOTIC METABOLISM
HALLMARK ADIPOGENESIS
HALLMARK BILE ACID METABOLISM
HALLMARK P53 PATHWAY
HALLMARK APICAL SURFACE
HALLMARK MYOGENESIS
HALLMARK HYPOXIA
HALLMARK GLYCOLYSIS
HALLMARK ANDROGEN RESPONSE
HALLMARK KRAS SIGNALING DN
HALLMARK IL2 STAT5 SIGNALING
HALLMARK HEME METABOLISM
HALLMARK CHOLESTEROL HOMEOSTASIS
HALLMARK APICAL JUNCTION
HALLMARK COAGULATION
HALLMARK KRAS SIGNALING UP
HALLMARK APOPTOSIS
HALLMARK UV RESPONSE DN
0 5 10 15 20 25
-log10 (P)

Figure S1

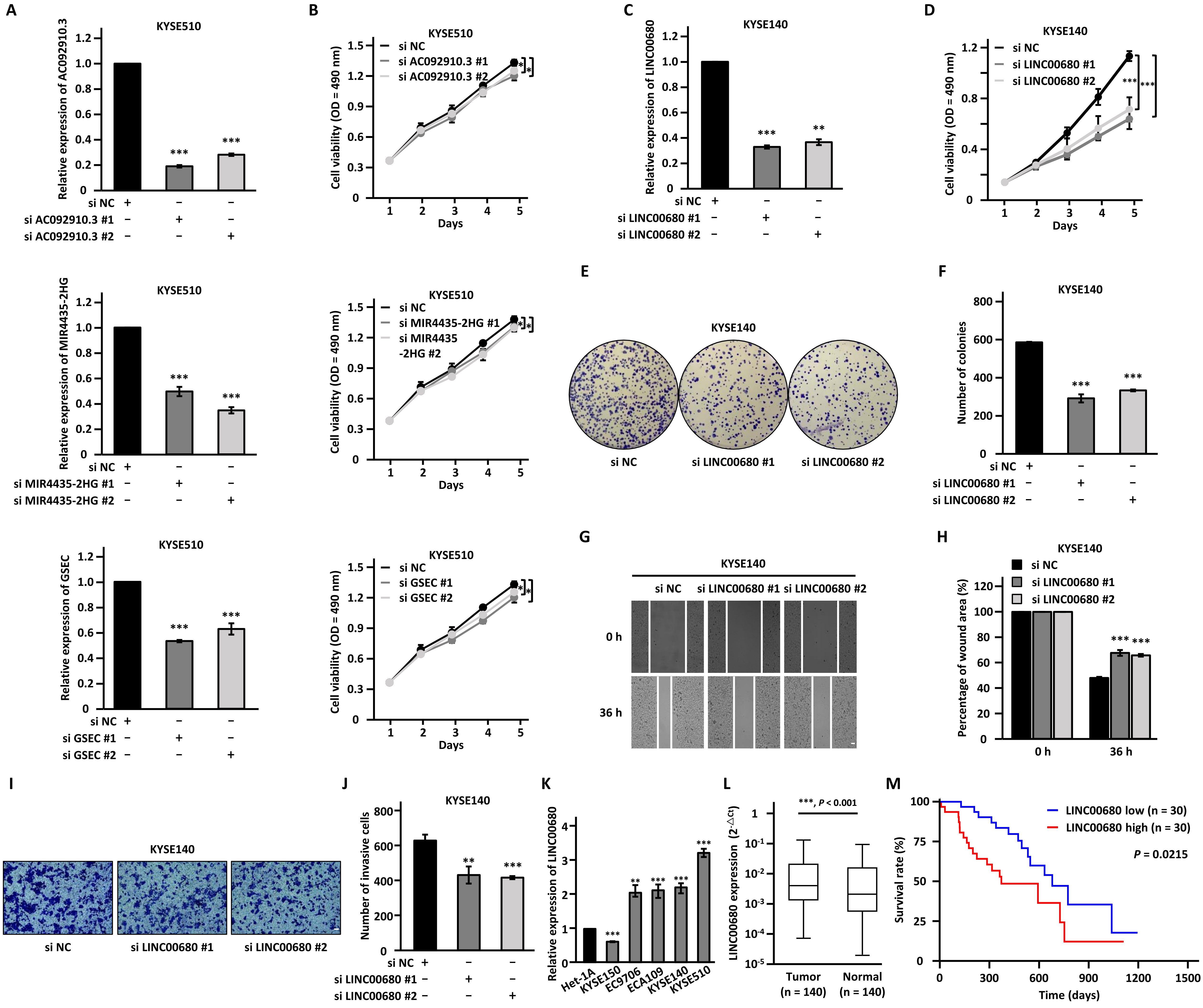

A
B
C
D
E
F
G
H
I
J
K
L
M
KYSE510
KYSE140
Relative expression of AC092910.3
Relative expression of MIR4435-2HG
Relative expression of GSEC
Relative expression of LINC00680
Cell viability (OD = 490 nm)
Days
si NC
si AC092910.3 #1
si AC092910.3 #2
si MIR4435-2HG #1
si MIR4435-2HG #2
si MIR4435
-2HG #2
si GSEC #1
si GSEC #2
si LINC00680 #1
si LINC00680 #2
Number of colonies
0 h
36 h
Percentage of wound area (%)
Number of invasive cells
Het-1A
KYSE150
EC9706
ECA109
KYSE140
KYSE510
***, P < 0.001
LINC00680 expression (2-△Ct)
Tumor
(n = 140)
Normal
(n = 140)
LINC00680 low (n = 30)
LINC00680 high (n = 30)
P = 0.0215
Survival rate (%)
Time (days)

Figure S2

A

Accession number	Coding Probability	Coding Label
NR_125727	0.0112573275436	no
NR_125728	0.0599273613537	no
NR_125729	0.0600814755457	no
NR_132994	0.0113950500378	no
NR_132995	0.0112877807112	no
NR_132996	0.0601775559813	no

B

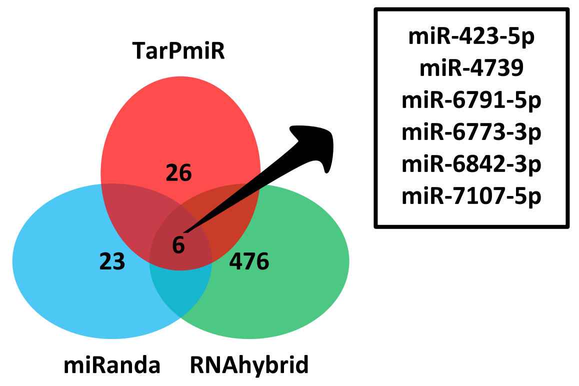

Figure S3

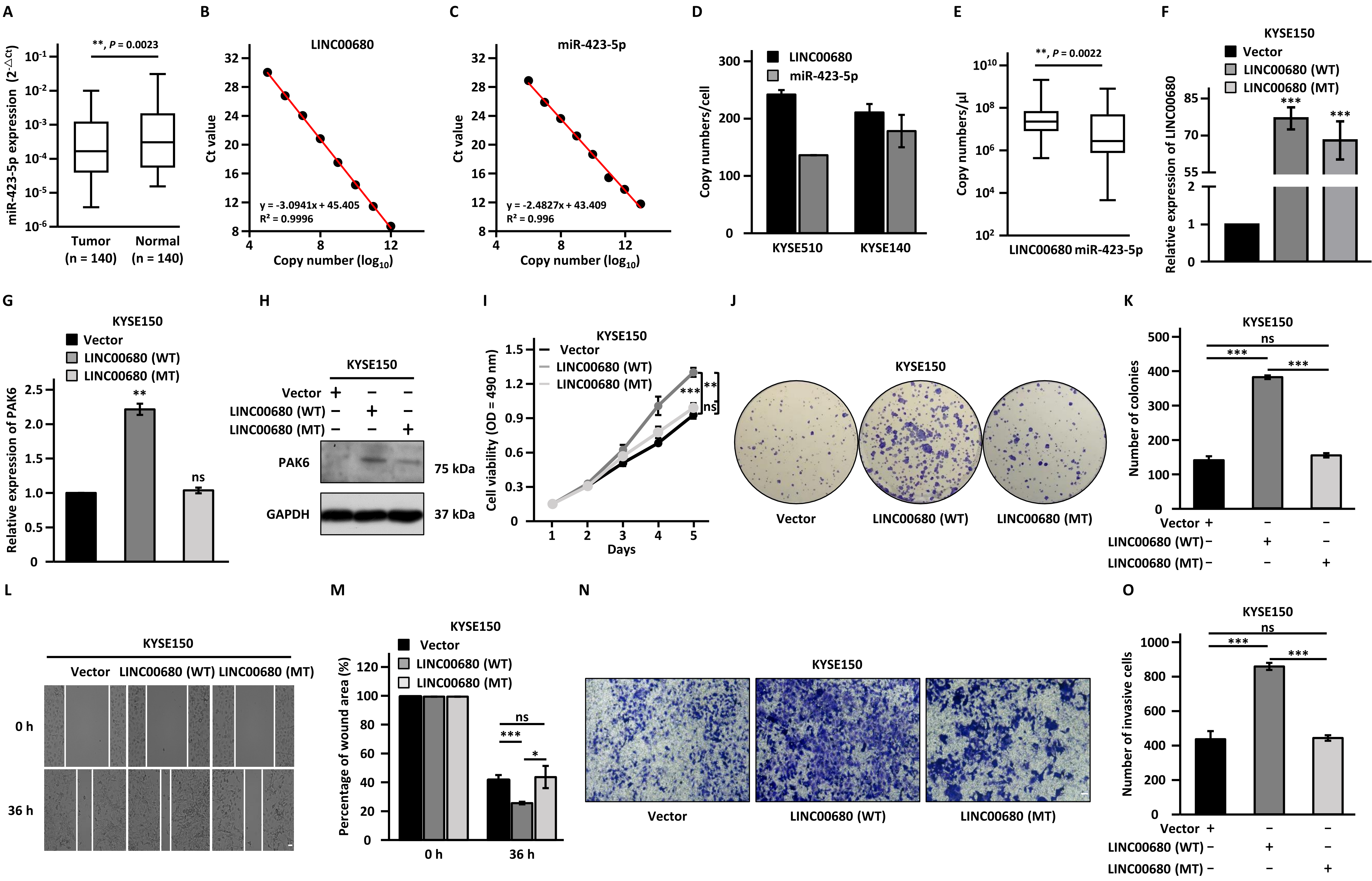
A
**, P = 0.0023
miR-423-5p expression (2-△Ct)
Tumor (n = 140)
Normal (n = 140)
B
LINC00680
Ct value
y = -3.0941x + 45.405
R² = 0.9996
Copy number (log10)
C
miR-423-5p
Ct value
y = -2.4827x + 43.409
R² = 0.996
Copy number (log10)
D
LINC00680
miR-423-5p
Copy numbers/cell
KYSE510
KYSE140
E
**, P = 0.0022
Copy numbers/μl
LINC00680
miR-423-5p
F
KYSE150
Vector
LINC00680 (WT)
LINC00680 (MT)
Relative expression of LINC00680
G
KYSE150
Vector
LINC00680 (WT)
LINC00680 (MT)
Relative expression of PAK6
H
KYSE150
Vector
LINC00680 (WT)
LINC00680 (MT)
PAK6
GAPDH
75 kDa
37 kDa
I
KYSE150
Vector
LINC00680 (WT)
LINC00680 (MT)
Cell viability (OD = 490 nm)
Days
J
KYSE150
Vector
LINC00680 (WT)
LINC00680 (MT)
K
KYSE150
Number of colonies
Vector
LINC00680 (WT)
LINC00680 (MT)
L
KYSE150
Vector
LINC00680 (WT)
LINC00680 (MT)
0 h
36 h
M
KYSE150
Vector
LINC00680 (WT)
LINC00680 (MT)
Percentage of wound area (%)
0 h
36 h
N
KYSE150
Vector
LINC00680 (WT)
LINC00680 (MT)
O
KYSE150
Number of invasive cells
Vector
LINC00680 (WT)
LINC00680 (MT)

Figure S4
